# Optimization of Crude Polysaccharide Extraction and Purification from the Black Outer Layer of the *Inonotus obliquus* Sterile Conk and Evaluation of the Purified Polysaccharide’s In Vitro Antioxidant Activity, Inhibitory Effects on Tumor Cell Viability, and Immunomodulatory Activity

**DOI:** 10.3390/foods15162901

**Published:** 2026-08-19

**Authors:** Miao Ding, Yang Su, Ruining Zhang, Hongxiang Liu, Yang Liu, Qi Wang

**Affiliations:** Engineering Research Center of Chinese Ministry of Education for Edible and Medicinal Fungi, College of Plant Protection, College of Mycology, Jilin Agricultural University, Changchun 130118, China; miaoding@mails.jlau.edu.cn (M.D.); 2192826870@mails.jlau.edu.cn (Y.S.); ruiningzhang@mails.jlau.edu.cn (R.Z.); hongxiangliu@jlau.edu.cn (H.L.)

**Keywords:** *Inonotus obliquus*, polysaccharides, response surface methodology, in vitro biological activities

## Abstract

*Inonotus obliquus* (*I. obliquus*) is a medicinal fungus rich in polysaccharides, polyphenols, and triterpenoids. However, studies on purified polysaccharide from the black outer layer of the *I. obliquus* sterile conk remain limited. In this study, crude polysaccharides were extracted by hot-water extraction, optimized using response surface methodology, and further purified by DEAE-52 ion-exchange chromatography and Sephacryl S-400 HR gel filtration chromatography. The structural characteristics and in vitro biological activities of the purified polysaccharide (IOP) were investigated. Under the optimized conditions, the crude polysaccharide extract yield reached 24.64 ± 0.48%. IOP contained 88.97 ± 1.33% total sugars and 10.91 ± 0.37% uronic acids, with a weight-average molecular weight of 78.0 kDa. Monosaccharide analysis revealed that IOP consisted of glucose, galactose, galacturonic acid, and glucuronic acid, and FT-IR analysis revealed the characteristic functional groups of polysaccharides. IOP exhibited concentration-dependent antioxidant activities, inhibited the cell viability of A549, SW1990, 4T1, and B16F10 cells with low cytotoxicity toward L929 fibroblasts, and stimulated the production of NO, TNF-α, IL-6, and IL-1β in RAW264.7 macrophages. These findings provide preliminary evidence that the purified IOP fraction obtained from the black outer layer of the *I. obliquus* sterile conk exhibits in vitro antioxidant, inhibitory effects on tumor cell viability, and immunomodulatory activities under the tested experimental conditions.

## 1. Introduction

Polysaccharides are macromolecules composed of monosaccharide units linked by glycosidic bonds and are widely distributed in plants, animals, and microorganisms [1,2]. Fungal polysaccharides have been reported to exhibit antioxidant, immunomodulatory, antitumor, and gut microbiota-regulating activities [3]. These polysaccharides hold significant potential for application in functional foods, health products and natural medicines [4].

*Inonotus obliquus* (*I. obliquus*) is a basidiomycete fungus belonging to the family Hymenochaetaceae and is rich in polysaccharides, polyphenols, triterpenoids, and melanin [5]. Polysaccharides are considered important bioactive components responsible for many biological activities [6]. During natural growth, *I. obliquus* forms a sterile conk (Chaga) characterized by a melanized black outer layer surrounding yellow-brown inner tissue. This outer layer contains abundant melanin, polyphenols, triterpenoids, and other bioactive compounds and may possess physicochemical characteristics distinct from those of the inner tissue [7]. Current research on *I. obliquus* polysaccharides (IOPs) mainly focuses on polysaccharides derived from whole sclerotia, fruiting bodies, or cultured mycelia [8]. In contrast, the black outer layer of the *I. obliquus* sterile conk has received considerably less attention as an independent material, particularly with respect to the optimization of polysaccharide extraction, purification, structural characterization, and in vitro biological activities. Therefore, systematic investigation of polysaccharides from this specific part may provide additional information on the chemical characteristics and biological potential of *I. obliquus*.

Unlike studies using whole sterile conks, fruiting bodies, or cultured mycelia, the present study specifically used the black outer layer of the sterile conk as the starting material for extraction optimization, purification, and subsequent characterization and in vitro bioactivity evaluation. Accordingly, this study aimed to systematically investigate the polysaccharide fraction from the black outer layer of the *I. obliquus* sterile conk. The crude polysaccharide-rich extract was first obtained by hot-water extraction, and the extraction conditions were optimized using single-factor experiments and response surface methodology. The resulting extract was subsequently purified by DEAE-52 ion-exchange chromatography and Sephacryl S-400 HR gel filtration chromatography. The purified polysaccharide fraction (IOP) was characterized in terms of its chemical composition, molecular weight, monosaccharide composition, and FT-IR characteristics. Finally, its in vitro antioxidant, inhibitory effects on tumor cell viability, and immunomodulatory activities were evaluated. This study provides a preliminary systematic evaluation of a relatively underexplored tissue of *I. obliquus* and establishes a basis for further structural and mechanistic investigations.

## 2. Materials and Methods

### 2.1. Materials

*I. obliquus* was supplied by the Center for Edible and Medicinal Fungi, Jilin Agricultural University, Changchun, China. Anhydrous ethanol, chloroform, n-butanol (Tianjin Xinbote Chemical Co., Ltd., Tianjin, China), phenol (Beijing Chemical Works, Beijing, China), and concentrated sulfuric acid (Xilong Science Co., Ltd., Shantou, China) were of analytical grade. A protein assay kit utilizing bicinchoninic acid (BCA) was obtained from Bioss Biotechnology Co., Ltd. located in Beijing, China. Additionally, the kits for assessing total polyphenol content and endotoxin detection were sourced from Beyotime Biotechnology Co., Ltd. in Shanghai, China. Shanghai Yuanye Bio-Technology Co., Ltd. (Shanghai, China) supplied fucose (Fuc), rhamnose (Rha), arabinose (Ara), galactose (Gal), glucose (Glc), xylose (Xyl), mannose (Man), fructose (Fru), galacturonic acid (GalA), and glucuronic acid (GlcA). The detection kits for 2,2-Diphenyl-1-picrylhydrazyl (DPPH), 2,2′-azino-bis (3-ethylbenzothiazoline-6-sulfonic acid) (ABTS), and hydroxyl radical (·OH) free radical scavenging were acquired from Nanjing Jiancheng Bioengineering Institute located in Nanjing, China. Kits for assessing superoxide anion (O_2_·^−^) radical scavenging ability and ferric ion reducing antioxidant power (FRAP) were sourced from Beijing Boxbio Biotechnology Co., Ltd. (BOXBIO) in Beijing, China. LONSERA premium South American fetal bovine serum (FBS) was obtained from Suzhou Shuangru Biotechnology Co., Ltd. in Suzhou, China. High-glucose DMEM and RPMI-1640 media were procured from Gibco in New York, NY, USA. Serum-free cell cryopreservation solutions were purchased from New Cell and Molecular Biotech, in Suzhou, China. Cellulose DEAE-52, KBr, Vitamin C (VC), penicillin–streptomycin mixture, trypsin digestion solution, phosphate-buffered saline (PBS), lipopolysaccharide (LPS) and Gemcitabine Hydrochloride were procured from Solarbio Biotechnology Co., Ltd. (Beijing, China). The HiPrep Sephacryl S-400 HR chromatography column was purchased from Cytiva Life Sciences (Shanghai) Co., Ltd., Shanghai, China. The Cell Counting Kit-8 (CCK-8, K1018) was acquired from APExBIO Company located in Houston, TX, USA. ELISA kits for measuring nitric oxide (NO), tumor necrosis factor-α (TNF-α), interleukin-6 (IL-6), and interleukin-1β (IL-1β) were sourced from Shanghai Preferred Biotechnology Co., Ltd. in Shanghai, China.

### 2.2. Optimization of the Extraction Process for IOPs

#### 2.2.1. Extraction

*I. obliquus* was authenticated by internal transcribed spacer (ITS) sequence analysis (GenBank: GU903006.1) (Figure S1). As shown in Figure S2, the black outer layer of the *I. obliquus* sterile conk was manually collected, dried, powdered, 60-mesh sieved and placed in beakers to serve as the raw material for extracting crude polysaccharides. RO water was added at the specified liquid-to-solid ratio, and the extraction was performed twice under the designated temperature and time conditions. The supernatants were then combined. After extraction, the suspension was centrifuged, and the resulting supernatant was concentrated and precipitated with ethanol (concentration of 75%, *v*/*v*), and the mixture was allowed to precipitate overnight at 4 °C. The precipitate was collected by centrifugation (7500 rpm, 5 min), dried, and weighed to determine the crude polysaccharide extract yield [9]. The crude polysaccharide extract yield was calculated based on the weight of the dried ethanol-precipitated material relative to the initial dry weight of the raw material, according to the following equation:

(1) Crude polysaccharide extract yield (%) = (weight of dried ethanol-precipitated extract/ initial dry weight of raw material) × 100%

#### 2.2.2. Single-Factor Experiment

The black outer layer of the *I. obliquus* sterile conk (2.0 g) was weighed. In the experiment focused on extraction time, the process was conducted with a liquid-to-solid ratio of 30:1 (mL/g) over durations of 60, 90, 120, 150, 180, and 210 min at a temperature of 80 °C. For the extraction temperature experiment, the conditions were set at a 30:1 (mL/g) ratio for 180 min, temperatures were 50, 60, 70, 80, 90, and 100 °C. In the liquid-to-solid ratio experiment conducted at 80 °C for 180 min, the ratios tested were 10:1, 20:1, 30:1, 40:1, 50:1, and 60:1 (mL/g). As outlined in Section 2.2.1, the crude polysaccharide extracts were obtained, and the extraction yield was calculated using Equation (1).

#### 2.2.3. Response Surface Methodology (RSM)

The crude polysaccharide extracts were optimized using Box–Behnken response surface analysis. Extraction temperature, liquid-to-solid ratio and time were selected as the independent variables, and the crude polysaccharide extract yield was used as the response variable. The coded levels of −1, 0, and 1 represented the low, center, and high levels of each variable, respectively (Table 1).

#### 2.2.4. Validation of the Optimized Extraction Conditions

The optimized extraction conditions predicted by the RSM model were experimentally validated using three independent experiments. The crude polysaccharide extract yield obtained under the optimized conditions was compared with the model-predicted value to evaluate the reliability and predictive accuracy of the model.

### 2.3. The Extraction, Isolation and Purification of IOP

The black outer layer of the *I. obliquus* sterile conk was extracted twice under optimized conditions in Section 2.2. Ethanol was added to the concentrated extract to obtain a final ethanol concentration of 75% (*v*/*v*), and the mixture was allowed to precipitate overnight at 4 °C. The precipitate was then subjected to centrifugation, after which it was redissolved and treated with Sevag reagent (n-butanol/chloroform, 1:4, *v*/*v*) repeatedly until no obvious protein precipitate was observed [10]. The solution underwent dialysis in RO water at 4 °C for a duration of 72 h, utilizing a 3500 Da membrane. The water was changed every 8 h. Subsequently, the crude *I. obliquus* polysaccharides (IOPs) were obtained by freeze-drying with a lyophilizer (SCIENTZ-12N/A, Ningbo, China). IOPs were further purified by DEAE-52 ion-exchange chromatography followed by HiPrep Sephacryl S-400 HR gel filtration chromatography. The major polysaccharide fraction obtained from DEAE-52 chromatography was further subjected to gel filtration chromatography. The major fraction was collected, dialyzed, and lyophilized to obtain the purified polysaccharide fraction, designated IOP, which was subsequently used for structural characterization and in vitro bioactivity evaluation.

### 2.4. Chemical Composition Analysis

The phenol–sulfuric acid method was used to assess the total sugar content. A bicinchoninic acid (BCA) kit was employed to determine the protein content. The sulfuric acid–carbazole method was used to determine the uronic acid content [11]. The content of phenolic compounds was determined using a commercial colorimetric assay kit and expressed as gallic acid equivalents (GAE). The contents of protein and phenolic compounds were evaluated within the detection ranges of the corresponding assay kits.

### 2.5. Endotoxin Detection

The endotoxin level of IOP was determined using a commercial endotoxin detection kit according to the manufacturer’s instructions. The assay was performed using an aseptic IOP solution prepared in endotoxin-free water.

### 2.6. Ultraviolet (UV)–Visible (Vis) Spectroscopy

A Cary 60 UV–Vis spectrophotometer (Agilent Technologies, Palo Alto, CA, USA) was used to record UV–Vis spectra ranging from 200 to 400 nm.

### 2.7. Determination of Molecular Weight

High-Performance Size-Exclusion Chromatography–Multi-Angle Laser Light Scattering–Refractive Index Detection (HPSEC-MALLS-RID) was used to determine the weight-average molecular weight (Mw) of IOP [12]. The analysis was performed using a Waters 2695 HPLC system (Waters Corporation, Milford, MA, USA) equipped with a TSK G5000PWXL column coupled with a TSK G4000PWXL column (Tosoh Corporation, Tokyo, Japan), a Wyatt DAWN HELEOS-II multi-angle laser light scattering detector (Wyatt Technology Corporation, Santa Barbara, CA, USA), and a Waters 2414 refractive index detector (Waters Corporation, Milford, MA, USA). Ammonium formate (50 mM) was used as the mobile phase at a flow rate of 0.5 mL/min, with the column temperature maintained at 35 °C and an injection volume of 50 μL.

### 2.8. Monosaccharide Composition Analysis

IOP was hydrolyzed with 2 M trifluoroacetic acid (TFA) at 120 °C for 2 h. The resulting hydrolysate was analyzed by high-performance anion-exchange chromatography with pulsed amperometric detection (HPAEC-PAD) to determine the monosaccharide composition [13].

### 2.9. Fourier Transform Infrared (FT-IR) Spectra

The FT-IR spectrum of IOP was recorded using a Nicolet™ iS50 spectrometer (Thermo, Waltham, MA, USA). The sample was mixed with KBr, pressed into a pellet, and scanned over the range of 4000–500 cm^−1^ [14].

### 2.10. In Vitro Antioxidant Activity Analysis

The antioxidant capabilities of IOP were evaluated through DPPH, ABTS, ·OH, O2·^−^ scavenging, and FRAP methods [15]. IOP was prepared in distilled water at concentrations of 0, 100, 200, 400, 800, and 1600 μg/mL, using VC as a positive control. The tests adhered to the manufacturer’s guidelines, and absorption was recorded at the specified wavelengths.

(2) Scavenging rate (%) = [(A_0_ − A_1_)/A_0_] × 100%

A_0_: OD (control); A_1_: OD (samples).

(3) Reduction capacity (μmol/mL) = **X** × *V*_total_/*V*_sample_ = 20 × **X**

where **X** (μmol/mL) represents the sample concentration calculated by substituting ΔA = A_1_ − A_0_ into the standard curve obtained according to the instructions provided with the commercial assay kit, y = 11.77x + 0.0039 (*R*^2^ = 0.9999). A_1_ represents the OD value of the sample, and A_0_ represents the OD value of the blank. *V*_total_ represents the total reaction volume (0.2 mL), and *V*_sample_ represents the total volume of sample added to the reaction system (0.01 mL).

### 2.11. In Vitro Inhibitory Effects on Tumor Cell Viability

Human lung cancer A549 cells, human pancreatic cancer SW1990 cells, mouse breast cancer 4T1 cells, and mouse melanoma B16F10 cells were obtained from iCell Bioscience Inc. located in Shanghai, China. The A549 and SW1990 cells were maintained in DMEM, while the 4T1 and B16F10 cells were cultured in RPMI-1640 supplemented with 10% FBS and 1% penicillin–streptomycin, at 37 °C with 5% CO_2_. Logarithmic-phase cells were introduced into 96-well plates (Guangzhou Jet Bio-Filtration Co., Ltd., Guangzhou, China) following three passages and were then incubated for 24 h. IOP at concentrations of 0, 100, 200, 400, 800, and 1600 μg/mL, along with 10 μM gemcitabine, were introduced for durations of 24, 48, and 72 h. Subsequently, CCK-8 was applied for 2 h, and the absorbance was measured at 450 nm [16].

(4) Inhibition rate (%) = {(A_c_ − A_s_)/(A_c_ − A_b_)} × 100%

A_s_: experimental group; A_c_: control group; Ab: blank group.

### 2.12. In Vitro Immunomodulatory Activity

RAW264.7 mouse monocyte–macrophage cells (iCell Bioscience Inc., Shanghai, China) were maintained in high-glucose DMEM supplemented with 10% FBS and 1% penicillin–streptomycin. RAW264.7 cells were treated with IOP at different concentrations for 24 h, with LPS (1 μg/mL) used as a positive control. Cell viability was then assessed using the CCK-8 assay according to the manufacturer’s instructions. After a 2 h incubation, the optical density was recorded at 450 nm [17].

(5) Cell viability (%) = {(A_s_ − A_b_)/(A_c_ − A_b_)} × 100%

A_s_: experimental group; A_c_: control group; A_b_: blank group.

Following a 24 h treatment with IOP, the supernatants were gathered, subjected to centrifugation, and then analyzed for NO, TNF-α, IL-6, and IL-1β using ELISA.

### 2.13. Statistical Analysis

Each experiment was carried out independently three times, with results expressed as the average ± standard deviation (SD). The analysis of the response surface was performed using Design-Expert 13.0. Statistical analyses were performed employing SPSS 26.0, and graphs were produced with GraphPad Prism 10.6.0.

## 3. Results and Discussion

### 3.1. Single-Factor Optimization

**Figure 1:**
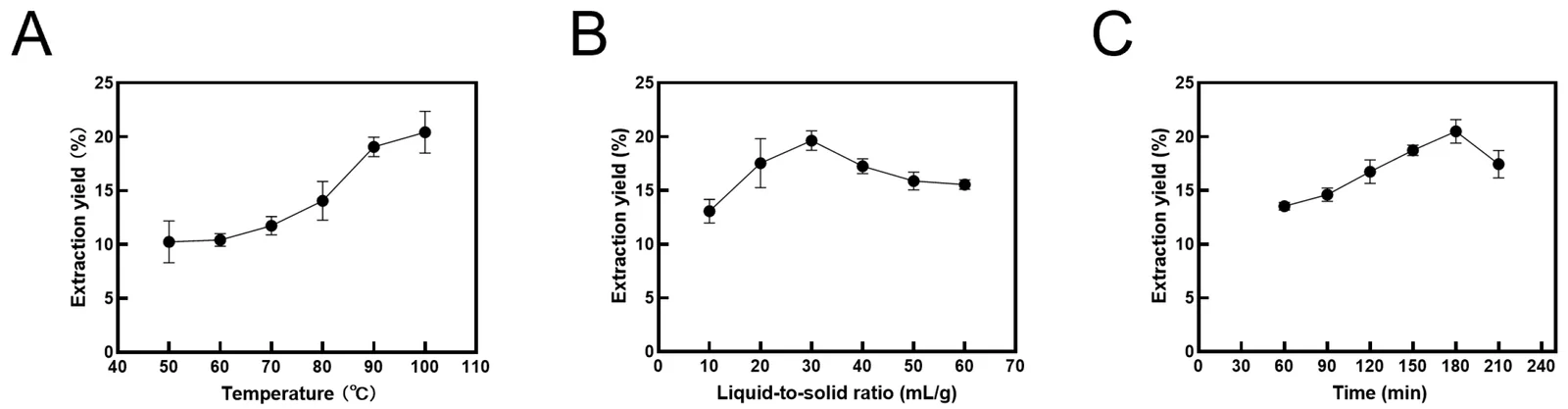
Effects of extraction parameters on the crude polysaccharide extract yield. (**A**) Extraction temperature. (**B**) Liquid-to-solid ratio of the extraction solution. (**C**) Extraction time.

From 50 to 100 °C, crude polysaccharide extract yield gradually increased, peaking at 100 °C (Figure 1A), likely due to enhanced solvent penetration, molecular diffusion, and polysaccharide dissolution. Similar trends have been reported in previous studies on polysaccharide extraction in other related extraction systems [18]. Because the difference in crude polysaccharide extract yield between 90 and 100 °C was relatively small, 90 °C was selected as the center point for the RSM experiment and the final optimum was determined by RSM. As shown in Figure 1B, increasing the liquid-to-solid ratio initially increased the crude polysaccharide extract yield, which reached 19.63 ± 0.90% at a ratio of 30:1 (mL/g). Further increases in the liquid-to-solid ratio did not improve the yield, possibly because excessive solvent addition did not further enhance polysaccharide release under the tested conditions [19]. Therefore, 30:1 (mL/g) was designated for subsequent optimization. As demonstrated in Figure 1C, the crude polysaccharide extract yield increased with time, reaching 20.49 ± 1.09% at 180 min. Beyond 180 min, the crude polysaccharide extract yield decreased, possibly because prolonged heating caused partial degradation or structural changes in polysaccharides [20]. Therefore, 180 min was selected for the subsequent experiments. The Box–Behnken experimental design and the corresponding experimental results are presented in Table 2.

### 3.2. Response Surface Methodology (RSM) Optimization

Design-Expert 13 (Stat-Ease Inc., Minneapolis, MN, USA) was used to perform regression analysis and response surface modeling [21]. The following regression equation was obtained:

(6) Y = 23.69 + 1.48A + 0.4416B + 0.1401C + 0.1026AB − 0.2198AC − 0.1503BC − 0.4321A^2^ − 2.77B^2^ − 1.06C^2^

**Figure 2:**
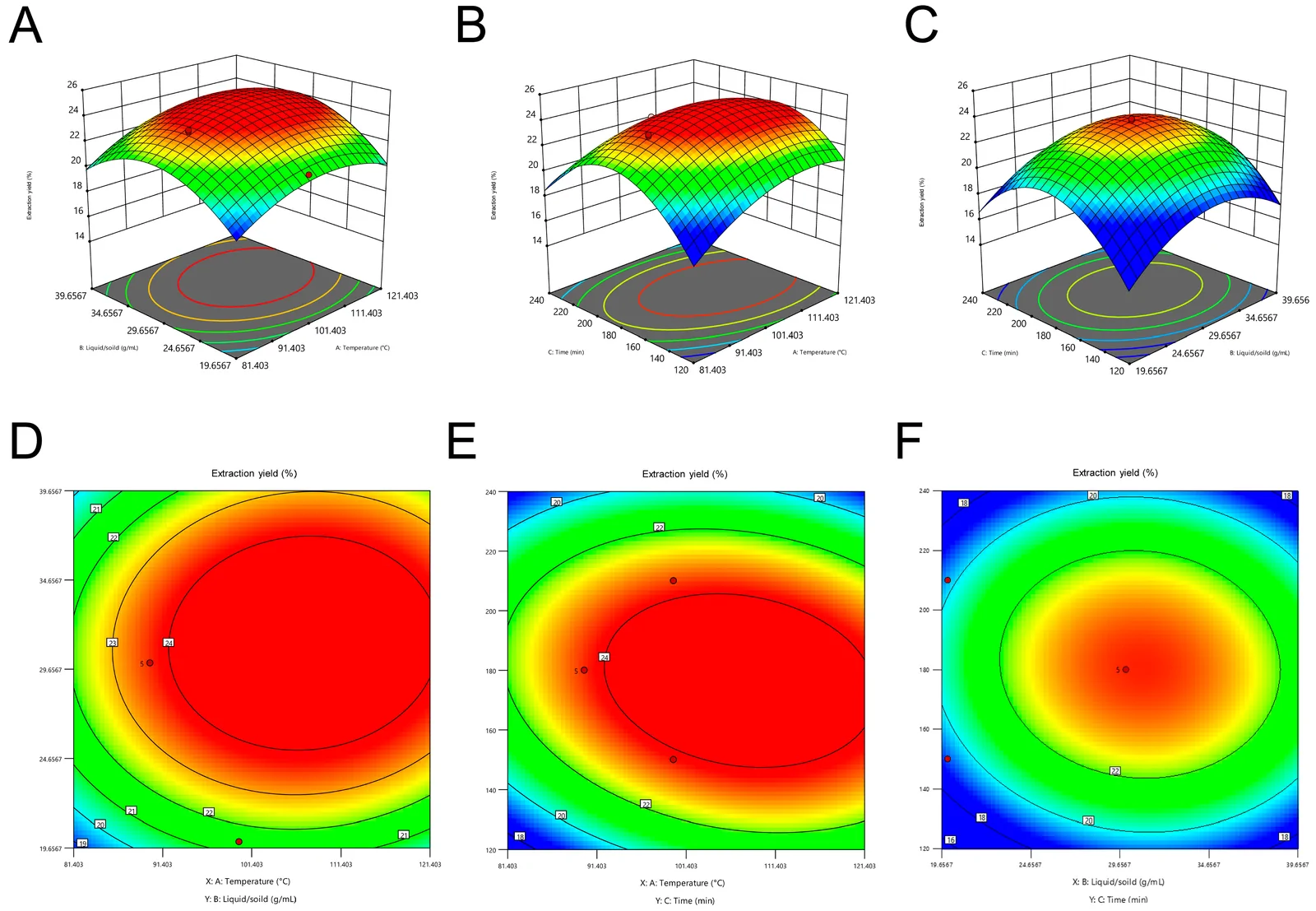
Response surface plots showing the effects of extraction variables on the crude polysaccharide extract yield. (**A**) Response surface plot showing the effect of extraction temperature and liquid-to-solid ratio on the crude polysaccharide extract yield. (**B**) Response surface plot showing the effect of extraction temperature and extraction time on the crude polysaccharide extract yield. (**C**) Response surface plot showing the influence of extraction liquid-to-solid ratio and extraction time on the crude polysaccharide extract yield. (**D**) Contour plot showing the influence of extraction temperature and extraction liquid-to-solid ratio on the crude polysaccharide extract yield. (**E**) Contour plot showing the influence of extraction temperature and extraction time on the crude polysaccharide extract yield. (**F**) Contour plot showing the influence of extraction liquid-to-solid ratio and extraction time on the crude polysaccharide extract yield.

ANOVA results for the regression model are shown in Table 3. The model demonstrated significance with a *p*-value of less than 0.0001, whereas the lack-of-fit was not significant, with a *p*-value of 0.6932, suggesting a good fit. High R^2^ (0.9978), adjusted R^2^ (0.9949), and low CV (0.6388%) demonstrated the reliability and accuracy of the regression model. The signs and values of the linear coefficients demonstrated the impact of each factor on the response value [22]. The linear effects of temperature (A), liquid-to-solid ratio (B), and extraction time (C) were statistically significant (*p* < 0.05), with temperature showing the strongest effect based on the F-values. According to the F-values, their effects followed the order A > B > C. Only the temperature-extraction time interaction (AC) was significant (*p* = 0.0156), indicating a significant interaction between temperature and extraction time. In contrast, AB (*p* > 0.05) and BC (*p* > 0.05) were not significant, indicating relatively weak temperature-liquid-to-solid ratio and liquid-to-solid ratio-extraction time interactions. The significant quadratic terms (A^2^, B^2^, and C^2^) indicated nonlinear relationships between the extraction variables and crude polysaccharide extract yield. Figure 2A–F shows 3D response and contour plots. The contour plots for the AB and BC interactions were nearly circular, indicating weak interactions between these variables, which was consistent with the ANOVA results. Conversely, the AC interaction’s contour plot displayed an elliptical shape, indicating a notable interaction between temperature and extraction time. Therefore, simultaneous optimization of these two variables was important for maximizing the crude polysaccharide extract yield.

The model forecasted the best conditions for extraction as follows: a temperature of 98 °C, a liquid-to-solid ratio of 32:1 (mL/g), and a duration of 173 min for the extraction process. Three experiments verified reliability. Predicted crude polysaccharide extract yield was 24.56%, and experimental crude polysaccharide extract yield was 24.64 ± 0.48% (*n* = 3), with a relative error of 0.33%, confirming that the developed RSM model accurately predicted the optimal processing conditions for crude polysaccharide extract yield [23].

**Table 3:** Results of analysis of variance for the regression model.

Source	Sum of Squares	Degrees of Freedom	Mean Square	F-Value	*p*-Value	Significance
**Model**	59.76	9	6.64	346.01	<0.0001	**
A—Temperature	17.54	1	17.54	913.88	<0.0001	**
B—Liquid/solid	1.56	1	1.56	81.28	<0.0001	**
C—Time	0.1571	1	0.1571	8.18	0.0243	*
AB	0.0421	1	0.0421	2.19	0.1821	
AC	0.1933	1	0.1933	10.07	0.0156	*
BC	0.0903	1	0.0903	4.71	0.0667	
A^2^	0.7860	1	0.7860	40.95	0.0004	**
B^2^	32.28	1	32.28	1681.73	<0.0001	**
C^2^	4.74	1	4.74	246.92	<0.0001	**
**Residual**	0.1343	7	0.0192			
Lack of Fit	0.0375	3	0.0125	0.5161	0.6932	not significant
Pure Error	0.0969	4	0.0242			
**Cor Total**	59.90	16				
**R^2^ = 0.9978**		**Adj.R^2^ = 0.9949**		**CV = 0.6388**		

Note: A, B, and C represent extraction temperature, liquid-to-solid ratio, and extraction time, respectively. AB, AC, and BC represent the interaction effects between the corresponding variables, while A^2^, B^2^, and C^2^ represent the quadratic effects. *p* < 0.05 indicates statistical significance (*), and *p* < 0.01 indicates high significance (**). R^2^ represents the coefficient of determination, Adj.R^2^ represents the adjusted coefficient of determination, and CV represents the coefficient of variation. A non-significant lack of fit indicates that the regression model adequately fits the experimental data.

### 3.3. Extraction, Isolation, Purification and Chemical Composition of IOP

**Figure 3:**
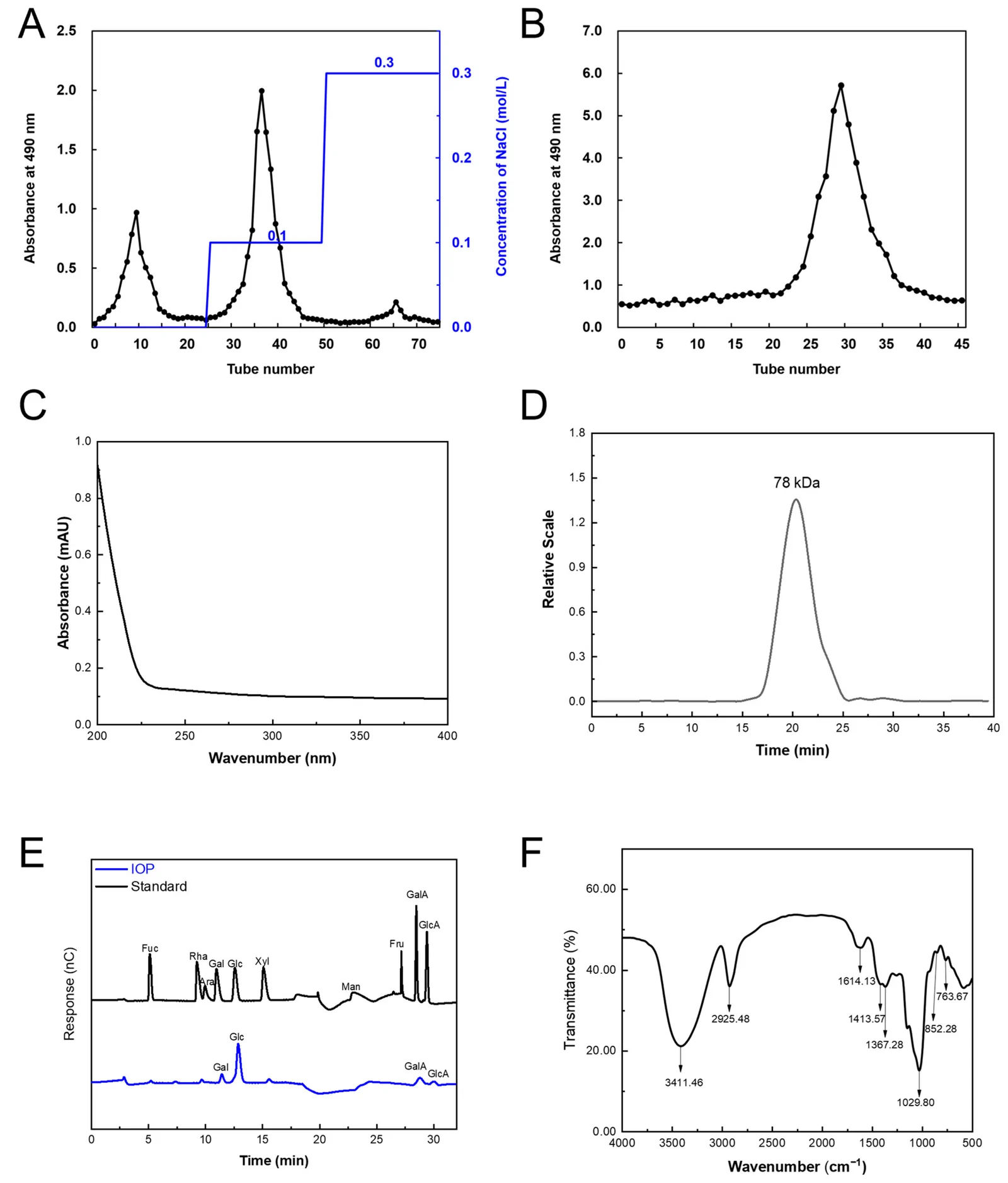
Isolation, purification, and structural characterization of IOP. (**A**) DEAE-52 ion-exchange chromatographic profile of the IOPs. (**B**) Sephacryl S-400 HR gel filtration chromatographic profile of the major fraction obtained from DEAE-52 chromatography. (**C**) UV–Vis spectrum of IOP. (**D**) Molecular weight peak of IOP. (**E**) Monosaccharide composition of IOP. (**F**) FT-IR spectrum of IOP. Note: UV–Vis, ultraviolet–visible; Mw, weight-average molecular weight; FT-IR, Fourier transform infrared spectroscopy.

Based on the optimized conditions, crude polysaccharide extracts were subsequently prepared under a temperature of 98 °C, a liquid-to-solid ratio of 32:1 (mL/g), and an extraction time of 173 min for further separation and purification. As shown in Figure 3A,B, IOPs were first separated by DEAE-52 ion-exchange chromatography. Among the fractions obtained from DEAE-52 chromatography, the fraction eluted with 0.1 M NaCl represented the predominant polysaccharide fraction and was therefore selected for further purification by HiPrep Sephacryl S-400 HR gel filtration chromatography. The purified polysaccharide was designated as IOP and subjected to subsequent structural characterization and in vitro bioactivity analysis. Chemical analysis revealed that IOP contained 88.97 ± 1.33% total sugars and 10.91 ± 0.37% uronic acids, with no detectable protein or phenolic compounds within the sensitivity ranges of the applied assay. No apparent absorption was observed in the UV–Vis spectrum of IOP at 260 and 280 nm (Figure 3C), further supporting the absence of detectable protein- and nucleic-acid-associated absorption.

### 3.4. Molecular Weight Determination and Monosaccharide Composition of IOP

As shown in Figure 3D, IOP exhibited a single symmetric elution peak, and its Mw was determined to be 78.0 kDa. Monosaccharide composition analysis revealed that IOP consisted of Glc, Gal, GalA, and GlcA, with molar percentages of 72.91%, 13.93%, 7.66%, and 5.50%, respectively (Figure 3E). The presence of GalA and GlcA further confirmed the existence of uronic acid residues in IOP, which was consistent with the physicochemical characterization results.

### 3.5. FT-IR Spectrum Analysis of IOP

The FT-IR spectrum of IOP exhibited several characteristic absorption bands associated with polysaccharide structures (Figure 3F). A broad absorption band at 3411.46 cm^−1^ was assigned to O–H stretching vibrations, while the band at 2925.48 cm^−1^ was attributed to C–H stretching vibrations [24]. The absorption bands at 1614.13 and 1413.57 cm^−1^ were mainly assigned to the asymmetric and symmetric stretching vibrations of carboxylate groups, respectively, which is consistent with the presence of uronic acid residues in IOP [14]. Although the absorption region around 1600–1650 cm^−1^ may overlap with the amide I region of proteins, no distinct additional amide-related bands were observed in the FT-IR spectrum. Moreover, the BCA assay did not detect protein within the detection range of the method, and no apparent absorption was observed at 280 nm in the UV–Vis spectrum. Therefore, the contribution of detectable proteinaceous components to the FT-IR spectrum appears to be minimal under the analytical conditions used. Nevertheless, the possibility of trace protein or glycoprotein components below the detection limit of the BCA assay cannot be completely excluded. Accordingly, the band at 1614.13 cm^−1^ was interpreted primarily as a carboxylate-associated vibration in the context of the chemical composition of IOP, rather than as direct evidence of peptide-derived amide groups. The band at 1367.28 cm^−1^ was assigned to C–H bending vibrations, while the absorption at 1029.80 cm^−1^ was associated with C–O–C and C–O–H stretching vibrations characteristic of polysaccharides [25]. Additional bands at 852.28 and 763.67 cm^−1^ were observed in the fingerprint region, further supporting the carbohydrate characteristics of IOP (Figure 3F). Collectively, the FT-IR spectrum exhibited characteristic absorption bands associated with polysaccharide structures and uronic acid residues, consistent with the chemical composition of IOP [26].

### 3.6. In Vitro Antioxidant Activity Analysis of IOP

**Figure 4:**
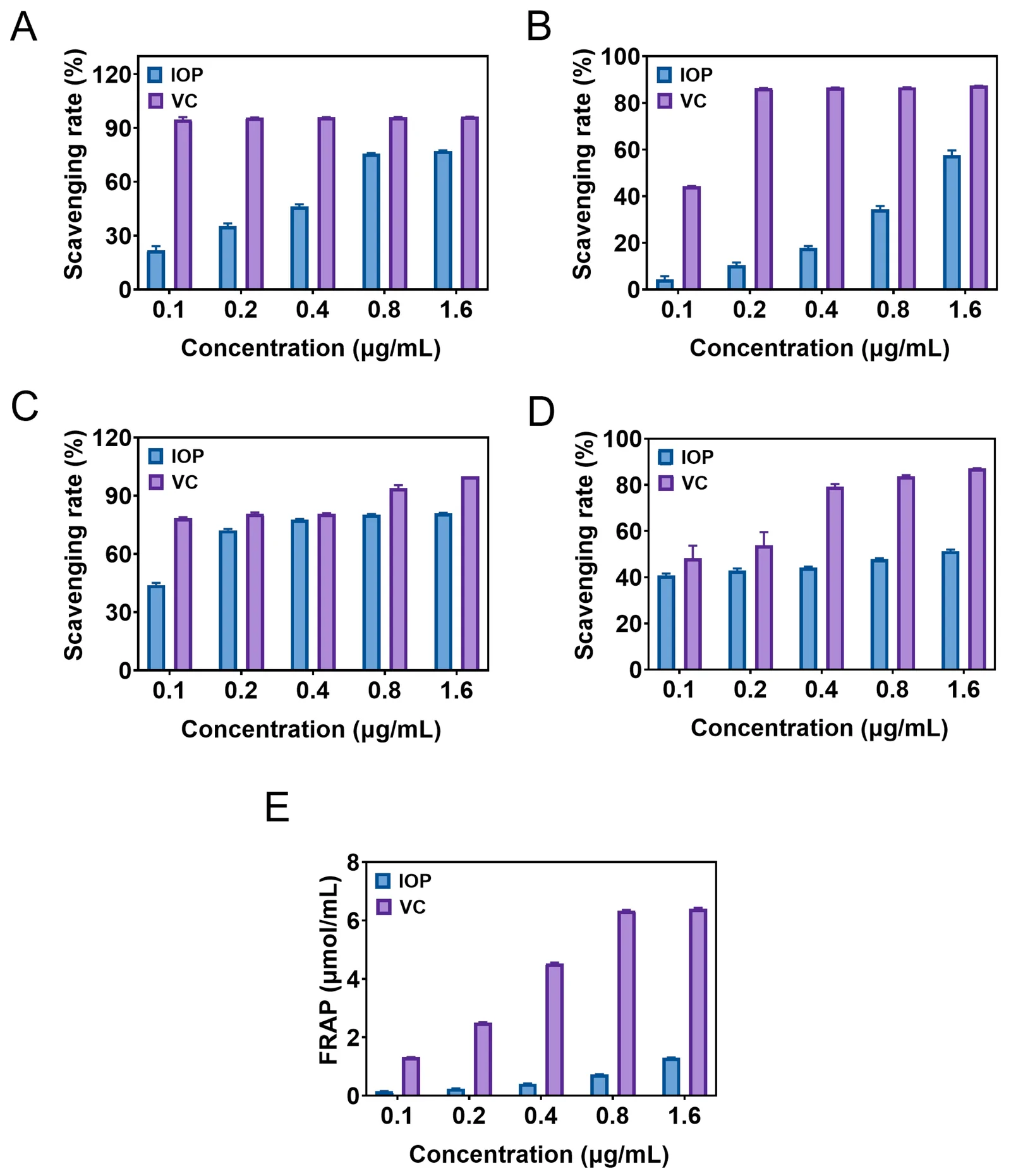
In vitro antioxidant activities of IOP at different concentrations. (**A**) DPPH radical-scavenging activity; (**B**) ABTS radical-scavenging activity; (**C**) hydroxyl radical (·OH)-scavenging activity; (**D**) superoxide anion (O_2_·^−^)-scavenging activity; and (**E**) ferric-reducing antioxidant power (FRAP). VC, vitamin C. Data are presented as mean ± SD (*n* = 3).

The connection between oxidative stress and processes such as aging, inflammation, and tumor formation is well-established. Natural polysaccharides have attracted considerable attention because of their diverse biological activities and generally favorable safety profiles [27]. However, the in vitro radical-scavenging activity of polysaccharides extracted from the black outer layer of the *I. obliquus* sterile conk has not been previously investigated. Figure 4 showed that IOP exhibited concentration-dependent scavenging activities against DPPH, ABTS, ·OH, and O_2_·^−^ radicals over the tested concentration range of 100–1600 μg/mL. Despite their lower activities than those of the positive control VC, IOP showed measurable radical-scavenging and ferric-reducing activities in vitro. At the maximum concentration tested (1600 μg/mL), the scavenging rates for DPPH, ABTS, ·OH, and O_2_·^−^ radicals were 77.24 ± 0.30%, 57.61 ± 2.01%, 80.97 ± 0.27%, and 51.33 ± 0.66%, respectively. Additionally, the ferric reducing antioxidant power (FRAP) value was 1.30 ± 0.02 μmol/mL. The IC_50_ values were further calculated to quantitatively evaluate the radical-scavenging activity of IOP, with the detailed results provided in Table S1.

Consistent with these findings, IOP exhibited concentration-dependent radical-scavenging and ferric-reducing activities under the tested in vitro conditions. Importantly, these antioxidant assays were conducted in cell-free chemical systems, in which the measured responses primarily reflect the interaction between IOP and the respective chemical radicals or redox systems rather than cellular osmotic stress. The concentration-dependent responses observed across multiple chemically distinct assay systems, together with the detectable ferric-reducing activity, support the presence of measurable chemical antioxidant activity of IOP under the tested in vitro conditions. However, the relatively high concentrations required to produce measurable effects, particularly for ABTS and O_2_·^−^ radical scavenging, indicate that these findings should be interpreted as in vitro chemical antioxidant activities rather than direct evidence of physiological antioxidant efficacy. Similar concentration-dependent antioxidant activities have also been reported for *I. obliquus* polysaccharides by Mu et al. [28]. Therefore, the present results provide evidence for the in vitro radical-scavenging and reducing capacity of IOP, while the physiological relevance of these chemical activities requires further investigation.

### 3.7. In Vitro Inhibitory Effects of IOP on Tumor Cell Viability

**Figure 5:**
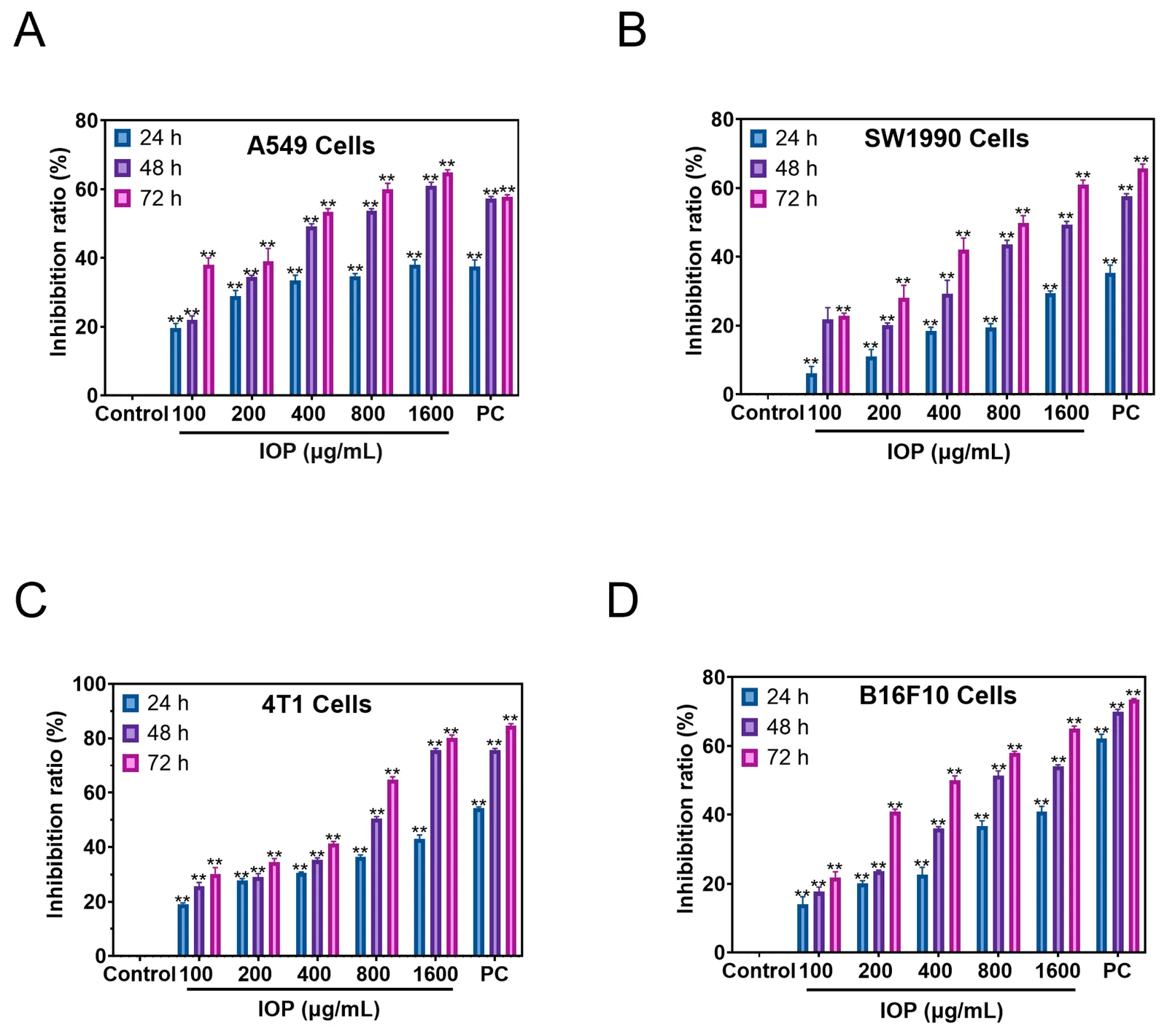
Inhibitory effects of IOP on tumor cell viability in vitro. (**A**) A549, (**B**) SW1990, (**C**) 4T1, (**D**) B16F10. Data were expressed as mean ± SD. ** *p* < 0.01 versus control group.

CCK-8 assays were used to assess the effects of IOP treatment on the cellular metabolic activity reflected by the CCK-8 readout under the tested conditions. As a preliminary approach for evaluating the biological activity of natural products, concentration- and time-dependent changes in CCK-8-derived viability-related responses are commonly assessed using this assay. As shown in Figure 5, IOP treatment resulted in concentration- and time-dependent decreases in the CCK-8-derived viability-related readout of A549, SW1990, 4T1, and B16F10 cells. The decrease in the CCK-8 readout was generally more pronounced with prolonged treatment, indicating a time-dependent response under the tested conditions. The calculated IC_50_ values are summarized in Table S2 and provide a quantitative description of the concentration- and time-dependent responses observed within the tested concentration range. For some tumor cell lines, the 24 h IC_50_ values exceeded the highest concentration tested (1600 μg/mL), indicating that the inhibitory effects were relatively weak at the shorter exposure time. Therefore, these results should not be interpreted as evidence of high-potency tumor cell inhibition, but rather as preliminary concentration- and time-dependent effects on the CCK-8-derived viability-related readout under the tested in vitro conditions.

The relatively high concentration range used in the present study warrants consideration of whether the addition of IOP could alter the osmotic environment of the cell culture system. IOP had a weight-average molecular weight of 78.0 kDa; therefore, the highest concentration tested (1.6 mg/mL) corresponds to approximately 0.0205 mmol/L of polymer molecules. Assuming ideal osmotic behavior and treating each polymer molecule as one osmotically active particle, the maximum theoretical osmotic contribution of IOP itself would be approximately 0.0205 mOsm/L. This contribution is negligible compared to the osmolality commonly used for mammalian cell culture, which is generally close to the physiological range of approximately 300 mOsm/kg [29]. Importantly, experimental studies have shown that elevated extracellular osmolality can affect mammalian cell proliferation, cell volume, and cellular metabolism, with the magnitude of these effects depending on the degree and duration of hyperosmotic exposure. For example, CHO cells exposed to 300, 370, 460, and 530 mOsm/kg exhibited progressively impaired cell growth and altered mitochondrial function at elevated osmolality [30]. In addition, hyperosmotic conditions have been shown to activate conserved cellular mechanisms involved in osmotic sensing and cell-volume regulation in mammalian cells [31]. Therefore, based on the molecular weight and concentration of IOP used in the present study, the IOP polymer itself would be expected to make only a negligible contribution to the osmotic environment and is unlikely, by itself, to account for the observed reduction in the CCK-8-derived readout through hyperosmotic stress. In addition, IOP was subjected to dialysis (3500 Da) before the subsequent structural and biological analyses, which would be expected to reduce the contribution of readily dialyzable low-molecular-weight solutes originating from the extraction and purification procedures. Nevertheless, because the osmolality of the final IOP-containing culture medium was not directly measured, a contribution from residual low-molecular-weight solutes cannot be completely excluded. Overall, the theoretical calculation indicates that the IOP polymer itself is unlikely to make a substantial contribution to the osmotic environment at the highest concentration tested. However, the relatively high concentrations used in this study may not be directly representative of physiologically achievable exposure levels. Accordingly, the observed effects should be interpreted as preliminary in vitro responses under the tested experimental conditions rather than definitive evidence of tumor cell death or a specific antitumor mechanism.

Meanwhile, IOP exhibited relatively low cytotoxicity toward L929 fibroblasts under the tested conditions (Figure S3). Within the concentration range of 100–1600 μg/mL, IOP did not cause a notable decrease in the CCK-8-derived viability-related readout of L929 cells after 24 or 48 h of exposure, and the readout remained above 89% at 800 and 1600 μg/mL after 72 h. In contrast, gemcitabine reduced the corresponding readout to less than 55% under the same conditions. These results indicate that IOP exhibited relatively low cytotoxicity toward L929 fibroblasts, while showing concentration- and time-dependent inhibitory effects on the CCK-8-derived viability-related readout of tumor cells under the tested conditions. However, because the CCK-8 assay primarily reflects cellular metabolic activity, it does not by itself establish the occurrence or mechanism of cell death. Therefore, the effects of IOP on the CCK-8-derived viability-related readout of tumor cells should be interpreted as preliminary in vitro findings, and further studies using complementary assays are required to determine whether these changes reflect effects on cell viability, proliferation, or other cellular processes and to elucidate the underlying mechanisms.

### 3.8. The Influence of IOP on the Viability and Immune Activity of RAW264.7 Cells

**Figure 6:**
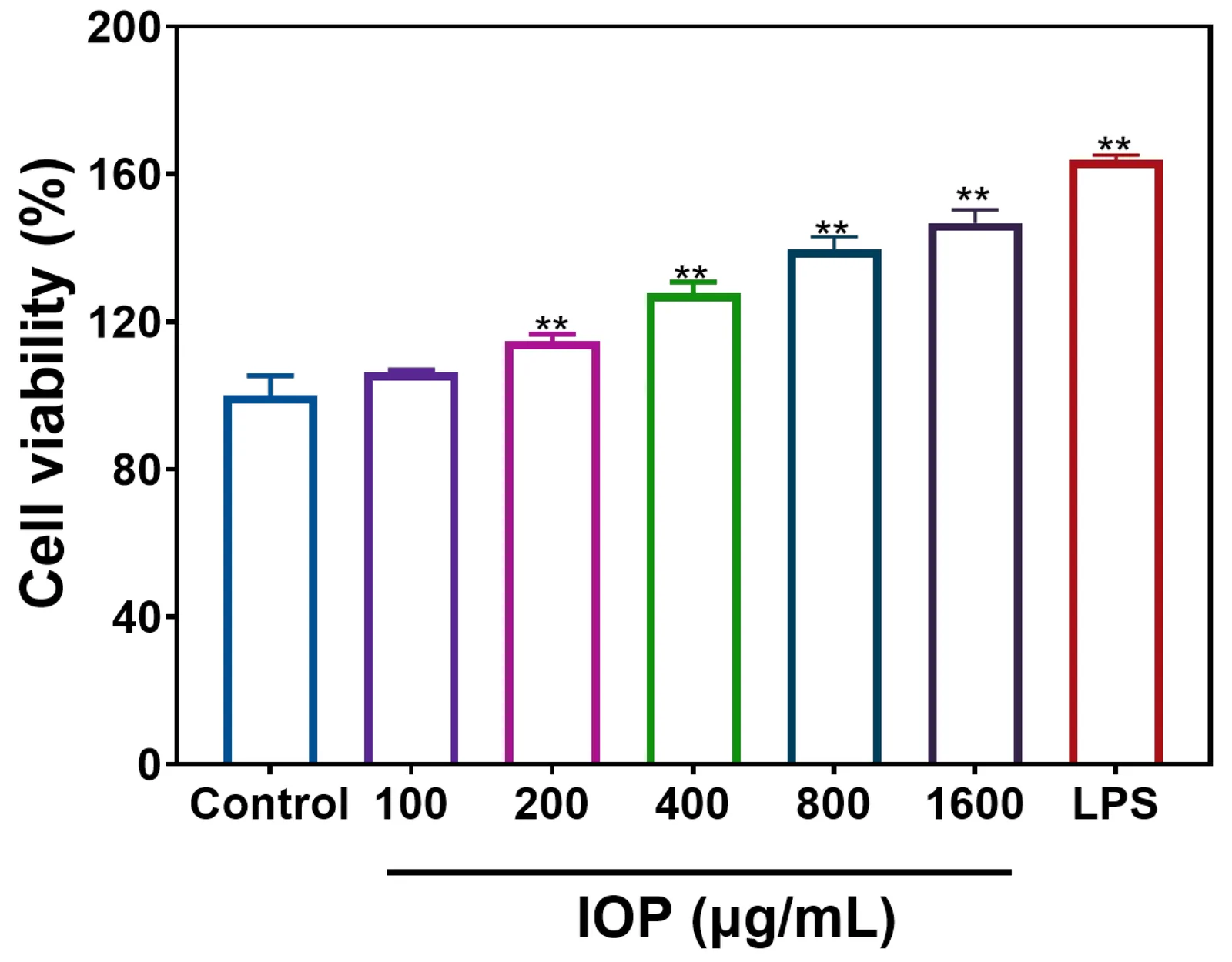
Effect of IOP on the viability of RAW264.7 cells in vitro. Data were expressed as mean ± SD. ** *p* < 0.01 versus control group.

**Figure 7:**
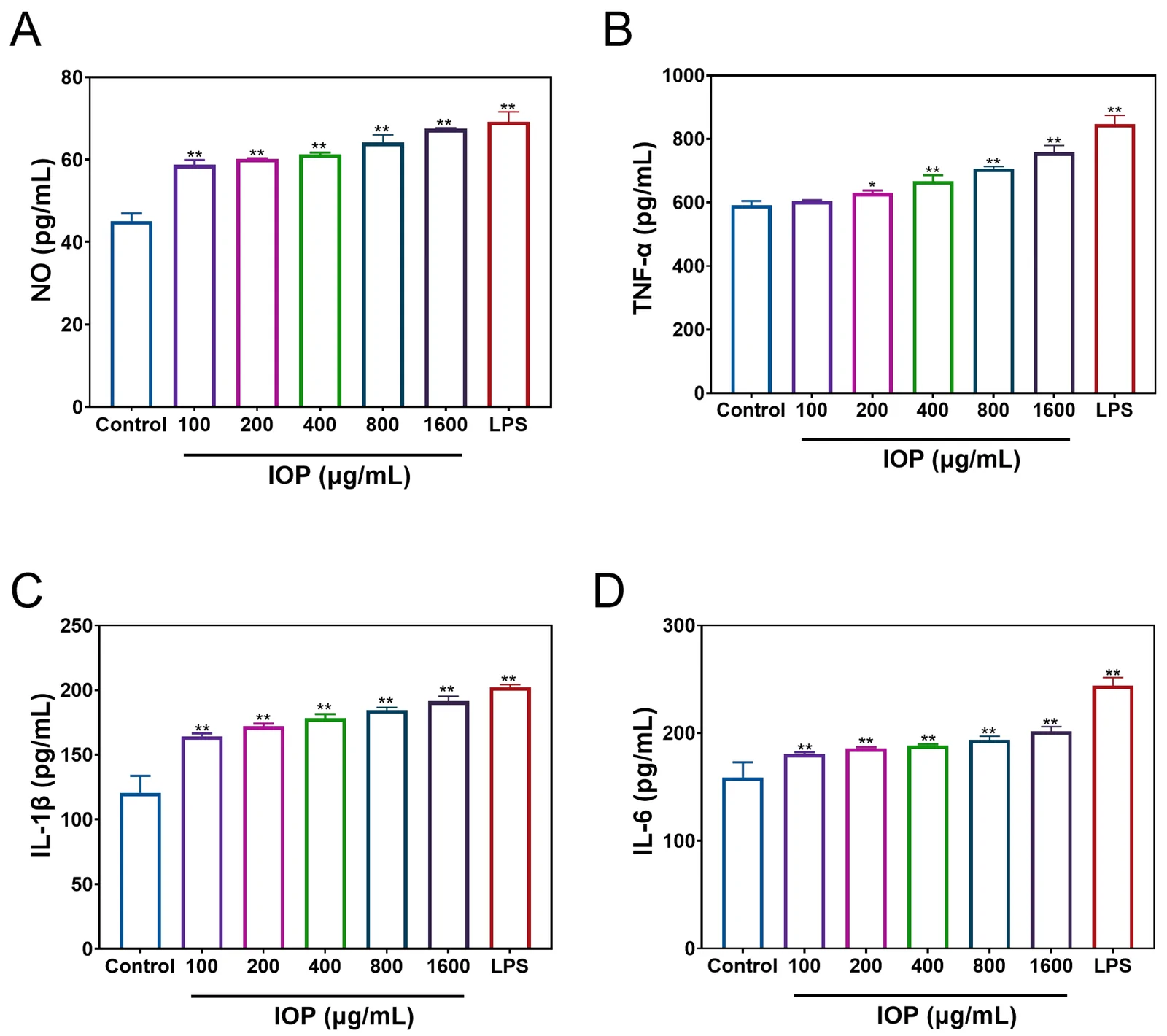
Effects of IOP on NO and cytokine production in RAW264.7 cells. (**A**) NO, (**B**) IL-6, (**C**) IL-1β, (**D**) TNF-α. Data were expressed as mean ± SD. * *p* < 0.05, ** *p* < 0.01 versus control group. NO, nitric oxide; IL-6, interleukin-6; IL-1β, interleukin-1β; TNF-α, tumor necrosis factor-α.

Macrophages are immune cells crucial for antigen recognition, inflammation regulation, and antitumor responses. Consequently, RAW264.7 cells are widely used as an in vitro model for evaluating macrophage-related immunomodulatory activity [32]. After 24 h of exposure to IOP at concentrations ranging from 100 to 1600 μg/mL, RAW264.7 cells showed no notable cytotoxic effects (Figure 6). Moreover, IOP substantially boosted the production of NO, IL-6, IL-1β, and TNF-α when compared to the control group (*p* < 0.01) (Figure 7), with secretion levels rising in accordance with the concentration. Although the effects were less pronounced than those of LPS, the responses increased with IOP concentration, indicating a concentration-dependent immunomodulatory effect in RAW264.7 cells [33].

To assess the potential contribution of endotoxin contamination to the immunomodulatory activity of IOP, the endotoxin level was determined. The endotoxin content of IOP was below the detection limit of the assay (<0.25 EU/mg). Based on this upper detection limit, treatment with IOP at the maximum concentration of 1600 μg/mL would correspond to a theoretical maximum endotoxin concentration of approximately 0.4 EU/mL in the treatment solution. Importantly, this value represents a worst-case upper bound rather than the measured endotoxin concentration, because the actual endotoxin content was below the detection limit. Nevertheless, because RAW264.7 macrophages are highly responsive to endotoxin, a contribution from trace endotoxin at the highest IOP concentrations cannot be completely excluded on the basis of the present endotoxin assay alone. Therefore, the increases in NO, TNF-α, IL-6, and IL-1β should be interpreted as immunomodulatory responses associated with IOP under the tested conditions, rather than as definitive evidence of endotoxin-independent macrophage activation. The present study did not include endotoxin-spiked controls, endotoxin-neutralization experiments, or TLR4-dependent mechanistic assays; therefore, the relative contribution of residual endotoxin versus the IOP fraction itself cannot be quantitatively distinguished. Future studies incorporating endotoxin-matched controls and/or endotoxin-neutralization approaches will be necessary to directly establish the endotoxin-independent immunomodulatory activity of IOP. NO and the measured cytokines are important mediators involved in macrophage activation and inflammatory signaling [34]. However, the present experiments were limited to the measurement of these immune-related mediators and therefore do not establish a specific signaling mechanism or direct antitumor immune effect of IOP.

## 4. Conclusions

In conclusion, the extraction process of crude polysaccharides from the black outer layer of the *I. obliquus* sterile conk was successfully optimized using response surface methodology, achieving an improved yield of the crude polysaccharide extract. The obtained crude polysaccharides were further purified by DEAE-52 ion-exchange chromatography and Sephacryl S-400 HR gel filtration chromatography to obtain a relatively homogeneous polysaccharide fraction (IOP). Structural characterization revealed that IOP had a weight-average molecular weight of 78.0 kDa and was mainly composed of glucose, galactose, galacturonic acid, and glucuronic acid, with typical polysaccharide functional groups identified by FT-IR analysis. Furthermore, IOP exhibited concentration-dependent antioxidant activities, inhibited the viability of A549, SW1990, 4T1, and B16F10 cells, and enhanced the production of NO, TNF-α, IL-6, and IL-1β in RAW264.7 macrophages. These findings provide preliminary evidence of the in vitro antioxidant, inhibitory effects on tumor cell viability, and immunomodulatory effects of the purified IOP fraction. However, the detailed structural features and underlying mechanisms remain to be elucidated, and further in vivo studies are required to determine the physiological relevance and biological significance of these effects.

## Figures and Tables

**Table 1:** The levels of factor in Box–Behnken design.

Factors	Code	Levels		
		−1	0	1
Temperature (°C)	A	80	90	100
Liquid-to-solid ratio (mL/g)	B	20:1	30:1	40:1
Time (min)	C	150	180	210

Note: A, B, and C represent extraction temperature, liquid-to-solid ratio, and extraction time, respectively. The coded levels −1, 0, and 1 represent the low, center, and high levels of each independent variable in the Box–Behnken design, respectively.

**Table 2:** Response surface methodology design and results from crude polysaccharide extracts of the black outer layer of the *I. obliquus* sterile conk.

	Factors			
Run No.	A: Temperature (°C)	B: Liquid/Solid (mL/g)	C: Time (min)	Extraction Yield (%)
**1**	100	30:1	210	23.60 ± 0.96
**2**	100	20:1	180	21.51 ± 0.54
**3**	100	40:1	180	22.47 ± 1.41
**4**	90	30:1	180	23.77 ± 0.52
**5**	90	30:1	180	23.65 ± 0.35
**6**	90	30:1	180	23.47 ± 0.80
**7**	90	40:1	210	20.34 ± 0.60
**8**	90	30:1	180	23.89 ± 0.48
**9**	80	20:1	180	18.71 ± 0.71
**10**	90	20:1	210	19.63 ± 0.26
**11**	80	40:1	180	19.27 ± 0.52
**12**	80	30:1	150	20.36 ± 0.64
**13**	100	30:1	150	23.72 ± 0.60
**14**	90	20:1	150	19.08 ± 0.47
**15**	90	40:1	150	20.39 ± 0.51
**16**	90	30:1	180	23.68 ± 0.33
**17**	80	30:1	210	21.12 ± 0.27

Note: A, B, and C represent extraction temperature, liquid-to-solid ratio, and extraction time, respectively. The crude polysaccharide extract yield was used as the response variable in the Box–Behnken design. Data are presented as mean ± standard deviation (*n* = 3).

## Data Availability

The original contributions presented in this study are included in the article/Supplementary Material. Further inquiries can be directed to the corresponding authors.

## Supplementary Materials

The following supporting information can be downloaded at https://www.mdpi.com/article/10.3390/foods15162901/s1, Table S1: IC_50_ values of IOP and vitamin C (VC) in different in vitro antioxidant assays; Table S2: IC_50_ values of IOP for different cell lines at different incubation times based on cell viability; Figure S1: ITS sequence alignment of the fungal sample; Figure S2: Photographs of the sterile conk of *I. obliquus* and its black outer layer; Figure S3: Effects of IOP on the viability of L929 fibroblasts.
