# Supplementary material for: Optimization of Crude Polysaccharide Extraction and Purification from the Black Outer Layer of the Inonotus obliquus Sterile Conk and Evaluation of the Purified Polysaccharide’s In Vitro Antioxidant Activity, Inhibitory Effects on Tumor Cell Viability, and Immunomodulatory Activity
Source: Foods. 2026 Aug 19;15(16):2901. doi: 10.3390/foods15162901 (PMC13512117; doi:10.3390/foods15162901)
Supplement: Supplementary file 1 [file foods-15-02901-s001.zip › foods-4466398-supplementary.pdf]

foods

Supplementary data

Optimization of crude polysaccharide extraction and purification from the black outer layer of the *Inonotus obliquus* sterile conk and evaluation of the purified polysaccharide's *in vitro* antioxidant activity, inhibitory effects on tumor cell viability, and immunomodulatory activity

Miao Ding [1], Yang Su [1], Ruining Zhang [1], Hongxiang Liu [1], Yang Liu [1,*] and Qi Wang [1,*]

[1] Engineering Research Center of Chinese Ministry of Education for Edible and Medicinal Fungi, College of Plant Protection, College of Mycology, Jilin Agricultural University, Changchun 130118, China; miaoding@mails.jlau.edu.cn (M.D.); 2192826870@mails.jlau.edu.cn (Y.S.); ruiningzhang@mails.jlau.edu.cn (R.Z.); hongxiangliu@jlau.edu.cn (H.L.)
* Correspondence: y_liu10@jlau.edu.cn (Y.L.); qiwang@jlau.edu.cn (Q.W.)

Table S1. IC_{50} values of IOP and vitamin C (VC) in different *in vitro* antioxidant assays.

Antioxidant assay	IC_{50} of IOP (μg/mL)	IC_{50} of VC (μg/mL)
**DPPH radical scavenging**	375.5	<100.0
**ABTS radical scavenging**	1289.0	100.2
**·OH scavenging**	< 100.0	<100.0
**$O_2^{\cdot-}$ scavenging**	1349.0	120.9

Note: IC_{50} values were calculated from concentration-dependent radical-scavenging curves and represent the concentration required to achieve 50% radical-scavenging activity.

Table S2. IC_{50} values of IOP for different cell lines at different incubation times based on cell viability.

Cell line	24 h	48 h	72 h
**A549**	>1600.0	611.8	408.5
**SW1990**	>1600.0	1599.0	770.0
**4T1**	>1600.0	616.2	409.0
**B16F10**	>1600.0	999.9	481.1

Note: IC_{50} values were calculated from concentration-dependent cell viability curves. Values >1600 μg/mL indicate that 50% inhibition was not reached at the highest tested concentration (1600 μg/mL).

Sequence ID: GU903006.1 Length: 822 Number of Matches: 1

Range 1: 62 to 822 GenBank Graphics ▼ Next Match

Score	Expect	Identities	Gaps	Strand
1376 bits(745)	0.0	757/762(99%)	3/762(0%)	Plus/Plus

```
Query  10   TATTTTG--ATCGAGGGGCCTGTGCTGGCACGGAAACGTTTGCATGTGCACGGCCTTTCG  67
            |||||||  |||||||||||||||||||||||||||||||||||||||||||||||||||
Sbjct  62   TATTTTGAAATCGAGGGGCCTGTGCTGGCACGGAAACGTTTGCATGTGCACGGCCTTTCG  121

Query  68   TGCTCAAATCCAACTCTCAAACCCCTGTGCACCTATACAAGTTGAAGGTCTTAGTAGTTT  127
            ||||||||||||||||||||||||||||||||||||||||||||||||||||||||||||
Sbjct  122  TGCTCAAATCCAACTCTCAAACCCCTGTGCACCTATACAAGTTGAAGGTCTTAGTAGTTT  181

Query  128  CTGTAATCGAACGGCAAGTCAAGTACGTCGAGTAATCAAGTACGAGGGTTTCGGCCCTTG  187
            ||||||||||||||||||||||||||||||||||||||||||||||||||||||||||||
Sbjct  182  CTGTAATCGAACGGCAAGTCAAGTACGTCGAGTAATCAAGTACGAGGGTTTCGGCCCTTG  241

Query  188  GAAAGTGTGAAAGATGGAAAGGCAAGCTTCAGAGACAAGGAGACGAAAAGCTTTTGGCTT  247
            ||||||||||||||||||||||||||||||||||||||||||||||||||||||||||||
Sbjct  242  GAAAGTGTGAAAGATGGAAAGGCAAGCTTCAGAGACAAGGAGACGAAAAGCTTTTGGCTT  301

Query  248  CATTACAAACACCAATGTAATTGTTATGTGAATGAAATGCTCCTTGTGGGCGATAATAAA  307
            |||||||||||||||| |||||||||||||||||||||||||||||||||||||||||||
Sbjct  302  CATTACAAACACCAATATAATTGTTATGTGAATGAAATGCTCCTTGTGGGCGATAATAAA  361

Query  308  TACAACTTTCAACAACGGATCTCTAGGCTCTCGCATCGATGAAGAACGCAGCGAAATGCG  367
            ||||||||||||||||||||||||||||||||||||||||||||||||||||||||||||
Sbjct  362  TACAACTTTCAACAACGGATCTCTAGGCTCTCGCATCGATGAAGAACGCAGCGAAATGCG  421

Query  368  ATAAGTAATGTGAATTGCAGAATTCAGTGAATCATCGAATCTTTGAACGCACCTTGCGCC  427
            ||||||||||||||||||||||||||||||||||||||||||||||||||||||||||||
Sbjct  422  ATAAGTAATGTGAATTGCAGAATTCAGTGAATCATCGAATCTTTGAACGCACCTTGCGCC  481

Query  428  CCTTGGTATTCCGAGGGGCATGCCTGTTTGAGTGTCATGTTAATCTCAAATCGCTCGTCT  487
            ||||||||||||||||||||||||||||||||||||||||||||||||||||||||||||
Sbjct  482  CCTTGGTATTCCGAGGGGCATGCCTGTTTGAGTGTCATGTTAATCTCAAATCGCTCGTCT  541

Query  488  ATTCTTAATTGAAGTGGCTTTCGATTTGGACTTGGAGGTTTTGCTGGCCCGGGCGACTTT  547
            ||||||| ||||||||||||||||||||||||||||||||||||||||||||||||||||
Sbjct  542  ATTCTTATTTGAAGTGGCTTTCGATTTGGACTTGGAGGTTTTGCTGGCCCGGGCGACTTT  601

Query  548  GGTTGCCCTTGGTTTGTCGGCTCCTCTCAAATACATTAGCTGGACTTTGGTTCGCGTTTA  607
            ||||||||||||||||||||||||||||||||||||||||||||||||||||||||||||
Sbjct  602  GGTTGCCCTTGGTTTGTCGGCTCCTCTCAAATACATTAGCTGGACTTTGGTTCGCGTTTA  661

Query  608  CGGTGTAATAATGTAATGTTCACTAAGACGCTTGCCTAACAAGTCTGCTTCTAATAGTCC  667
            ||||||||||||||||||||||||||||||||||||||||||||||||||||||||||||
Sbjct  662  CGGTGTAATAATGTAATGTTCACTAAGACGCTTGCCTAACAAGTCTGCTTCTAATAGTCC  721

Query  668  TTAAGTTGGACAAGGATCCCTTCGTTGGGCCTTCTTGACACCTTTGACCTCAAATCAGGT  727
            ||||||||||||||||||||||||||||||||||||||||||||||||||||||||||||
Sbjct  722  TTAAGTTGGACAAGGATCCCTTCGTTGGGCCTTCTTGACACCTTTGACCTCAAATCAGGT  781

Query  728  AGGATTACCCGCTGAACTTAAGCATATCAATAAGGCGGAGGA  769
            |||||||||||||||||||||||||||||||||| |||||||
Sbjct  782  AGGATTACCCGCTGAACTTAAGCATATCAATAAG-CGGAGGA  822
```

Figure S1. ITS sequence alignment of the fungal sample. The ITS sequence showed >99% sequence similarity to *Inonotus obliquus* (GenBank accession No. GU903006.1), supporting its identification as *I. obliquus*.

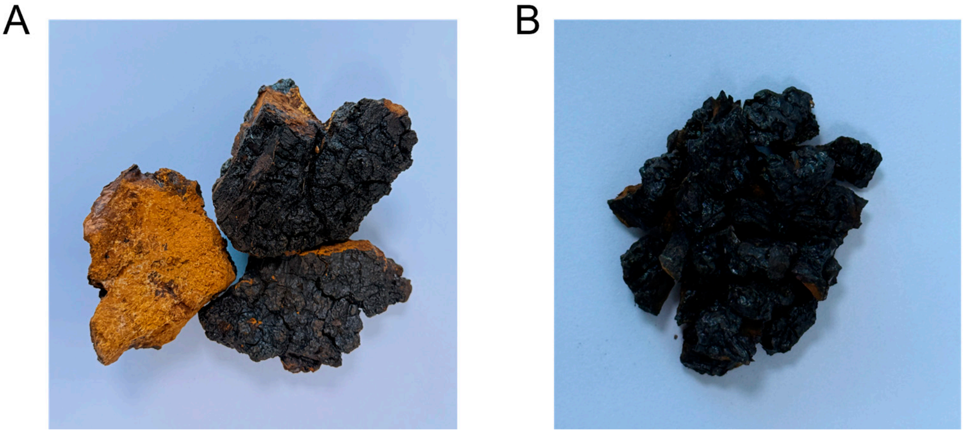

Figure S2. Photographs of the sterile conk of *I. obliquus* and its black outer layer. (A) Sterile conk of *I. obliquus*. (B) Black outer layer collected from the surface of the sterile conk and used as the raw material for polysaccharide extraction.

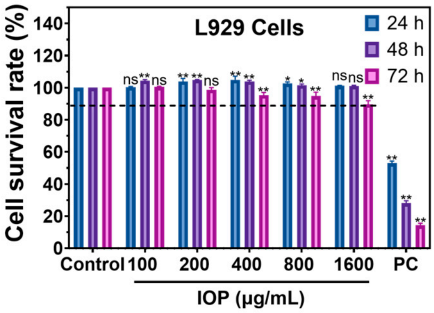

Figure S3. Effects of IOP on the viability of L929 fibroblasts. * $P < 0.05$, ** $P < 0.01$ versus control group.
